# Selection by Lambs Grazing Plantain (*Plantago lanceolata* L.), Chicory (*Cichorium intybus* L.), White Clover (*Trifolium repens* L.), Red Clover (*Trifolium pratense* L.) and Perennial Ryegrass (*Lolium perenne* L.) across Seasons

**DOI:** 10.3390/ani10122292

**Published:** 2020-12-03

**Authors:** Sharini C. Somasiri, Paul R. Kenyon, Patrick C.H. Morel, Stephen T. Morris, Peter D. Kemp

**Affiliations:** 1 Department of Animal and Food Sciences, Faculty of Agriculture, Rajarata University of Sri Lanka, Puliyankulama, Anuradhapura 50000, Sri Lanka; sharinisc@yahoo.com; 2 International Sheep Research Centre, School of Agriculture and Environment, Massey University, Palmerston North 4422, New Zealand; p.r.kenyon@massey.ac.nz (P.R.K.); P.C.Morel@massey.ac.nz (P.C.H.M.); S.T.Morris@massey.ac.nz (S.T.M.)

**Keywords:** selective grazing, diverse pastures, plantain, chicory, red clover, white clover, sheep

## Abstract

**Simple Summary:**

Diverse pastures based on combinations of the forage herb species plantain and chicory with red clover and white clover, provide superior annual feeding value to finish lambs in temperate regions, compared to the traditionally used perennial ryegrass and white clover pastures. The success of herb-clover diverse pastures is dependent on maintaining the relative abundance of each species in the mix over time. The selective grazing by lambs on herb-clover mixes was compared with their selection on perennial ryegrass and white clover pasture, by using randomly tagged individual plants, to determine if selective grazing changed the relative abundance of any of the herb or clover species. Variations in the selection of the forage species over the seasons of the year were observed, but best practice grazing management was shown to maintain the relative abundance of species in herb-clover pastures required for superior lamb live weight gains.

**Abstract:**

Plantain (*Plantago lanceolata*) and chicory (*Cichorium intybus*) are now widely used in combination with clover species to provide greater annual lamb live weight gains than perennial ryegrass and white clover pasture. Reported selective grazing of the species in herb-clover mixes could potentially detrimentally change the relative abundance of species and decrease lamb production. Lambs were offered three herbage treatments: Pasture (perennial ryegrass and white clover) mix, plantain (plantain, red clover and white clover) mix and a chicory (chicory, plantain, red clover and white clover) mix in each of four seasons for two years. The experiment was a randomized complete block design with three replicates with 18–30 lambs per treatment replicate depending on the season. Lambs were rotationally grazed and fed ad libitum. Selection by the lambs of individually tagged plants within the pasture treatments was observed for three days on two occasions per season. Red clover was the most selected species on day 1, but by day 3 there was no difference in the selection of the species (*p* < 0.05). Plantain and chicory in the plantain and chicory mixes were selected less on day 1 in autumn relative to the other seasons (*p* < 0.05). It was concluded that three days of grazing before moving lambs maintained the relative abundance of species in the herb-clover mixes.

## 1. Introduction

Plantain and chicory have increasingly been used by farmers in various combinations with clover species to provide diverse, or mixed species, swards that improve livestock growth rates compared to perennial ryegrass and white clover swards from spring to autumn [1]. Swards with a diversity of species are liable to have the relative abundance of the species change in response to diet selection by livestock, and, consequently, not deliver the herbage or livestock production expected from the initial sward [2,3]. Therefore, grazing management that sustains the required balance of all species in herb and clover sward mixes needs to consider the diet selection by livestock of the individual species in the mixes [2,3].

Current best practice grazing advice for plantain and chicory is a minimum grazing height of 70 mm under rotational grazing to obtain maximum sheep and cattle growth rates [1,4], but the effect of this management on the relative abundance of species in a mix of the herb species and clover species is not known. In a mixed sward, parameters such as sward height, botanical composition, nutritive value, plant morphology, stage of growth and palatability of herbage and previous experience [2,3,5,6] all affect an animal’s selection of a given herbage. Further, the choice of species selected on the first day in a grazing area can alter the species composition in a mixed sward and, therefore, the choices available on the subsequent grazing days [2]. Thus, over time diet selection can change.

There is considerable literature on diet selection and preference with ryegrass/white clover swards [2,3,5,6,7]. If given a choice, sheep proportionally eat more white clover than ryegrass [8]. However, there is a lack of information on selection or preference, in herb-clover mixes. Pain et al. [9] observed that lambs grazed more red clover than chicory followed by plantain in a preference study with side by side monocultures of plantain, chicory and red clover under field conditions. Further, the preference for plantain was greater in late autumn than in early autumn. Similar results were observed in a diet selection study undertaken using a mixed sward of plantain, chicory and red clover where lambs selected red clover over chicory followed by plantain [10]. Pain et al. [11] in an indoor study, reported that the preference for both red clover and chicory was higher than for both plantain and ryegrass. Cave et al. [12] examined diet selection by lambs within a herb-clover mix of plantain, chicory, white- and red-clover during four seasons. They used the same lambs throughout their study and grazed the lambs for up to seven days each time on the herb-clover mix before returning the lambs to a ryegrass and white clover pasture. Cave et al. [12] observed that lambs generally preferentially selected red clover over plantain and chicory from late spring to autumn, but concluded further research was required on whether this preferential selection affected the botanical composition of herb-clover swards. Combined, these studies indicate that lambs select red clover, white clover and chicory over plantain, which could lead to plantain becoming increasingly dominant in a herb-clover sward mix during its three to five year lifetime.

A consequence of plantain dominating in a herb-clover sward would probably be a decrease in lamb growth rates due to the feeding value of the sward declining. Previous studies based on the growth of lambs have shown that the feeding value of white clover is superior to chicory which in turn is superior to red clover, while ryegrass has a lower feeding value [13]. The feeding value of plantain has not been directly examined, but results from field experiments where plantain was grazed ad libitum by lambs suggests it is similar to, or higher than, that of a perennial ryegrass, white clover pasture mix [14]. Plantain can have higher fibre content [15] and lower digestibility than chicory [16], which suggests a lower feeding value than chicory. In addition, the ME content in both white- and red-clover is generally higher than that for plantain [17,18].

In this context, the following experiment was undertaken across four different seasons (early spring, late spring, summer and autumn) with lambs that had no previous diet experience with plantain and chicory and likely little experience with red clover. The objective was to see if there was preferential diet selection by lambs being rotationally grazed on herb-clover mix swards that changed the relative abundance of the species in the mix. Three herbage treatments consisting of pasture (perennial ryegrass and white clover) mix, plantain (plantain, red clover and white clover) mix and chicory (chicory, plantain, red clover and white clover) mix were examined with two hypotheses. The first hypothesis was that the most selected species in the herbage treatments would be white clover and red clover, and that this selection would be maintained across the different seasons. The second hypothesis was that plantain in the plantain mix and chicory mix would be selected at a lower proportion than the other species and that this low selection would be consistent in all four seasons.

## 2. Materials and Methods

### 2.1. Study Site

This experiment was undertaken using three herbage treatments from September 2011 to May 2012 at the Moginie Pasture and Crop Research Unit, Massey University, 4 km south of Palmerston North, New Zealand (40°21′ S and 175°37′ E, 30 m above sea level.). The experiment was sited on 6.75 ha of flat land. The soil type is Tokomaru silt loam, a Pallic soil, which although artificially drained is prone to waterlogging in winter and drying out in summer. The study had the approval of the Massey University Animal Ethics Committee (MUAEC 10119).

The herbage treatments were (i) ‘pasture mix’; perennial ryegrass (*Lolium perenne* L.) cultivar One50 and white clover (*Trifolium repens* L.) cultivar Grasslands Bounty; (ii) ‘plantain mix’; plantain (*Plantago lanceolata* L.) cultivar Ceres Tonic, white clover and red clover (*T. pratense* L.) cultivar Grasslands Sensation; and (iii) ‘chicory mix’; plantain, chicory (*Cichorium intybus* L.) cultivar Puna II, white- and red-clover.

**Figure 1:**
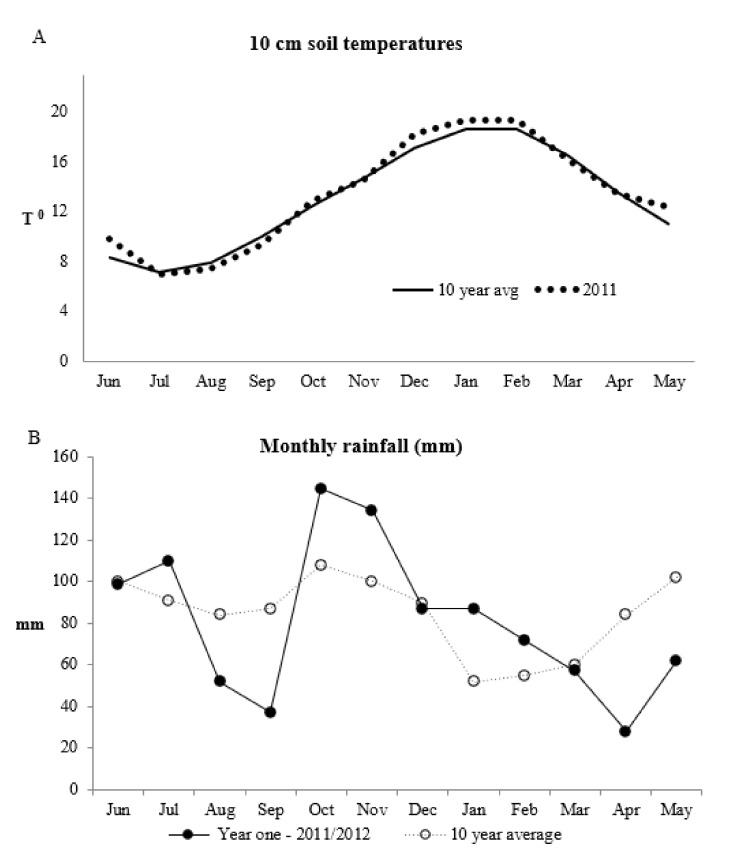
(**A**) Soil temperature (°C) and (**B**) rainfall (mm) at the experimental site for 2011 and the 10 year average.

The pasture mix was established by sowing perennial ryegrass and white clover at a rate of 16 kg/ha and 4 kg/ha respectively in 2009. Plantain mix and chicory mix were established in two steps. First, in autumn 2011 existing plantain pasture paddocks to be established as plantain mix and chicory mix were power harrowed to remove 1/3 of the existing plantain plants, established in 2009. Afterwards, plantain 6 kg/ha, white clover 4 kg/ha and red clover 6 kg/ha were sown to create the plantain mix, and chicory 6 kg/ha, plantain 6 kg/ha, white clover 4 kg/ha and red clover 6 kg/ha to create the chicory mix. The soil temperature (°C) and rainfall data (mm) at the experimental site are in Figure 1.

Source: National Institute of Water and Atmospheric Research (NIWA), AgResearch, Palmerston North, New Zealand. Weather station site was situated between 40°38′ S and 175°61′ E and the network number is E0536D.

The following fertilizers were applied to all paddocks; (i) 30% Potash Super (phosphorous 6.3%, potassium 15%, sulphur 7.7% and calcium 14%) basal fertilizer mixture (Ravensdown Ltd, Christchurch, New Zealand) at a rate of 400 kg/ha in autumn 2011; (ii) urea (Ravensdown, New Zealand) at a rate of 67 kg/ha (30 kg nitrogen/ha) in August and December 2011; (iii) Cropmaster 13 (Ravensdown, New Zealand) in June 2011 at a rate of 200 kg/ha as a source of nitrogen (12.6%), phosphorous (14%) and potassium (15%); and (iv) Cropmaster 15 in May 2012 at a rate of 200 kg/ha as a source of nitrogen (15.1%), phosphorous (10%), potassium (10%) and sulphate (7.7%) [19].

Details of the management of the pastures and lambs during the early spring [20], late spring and early summer [21], summer [22] and autumn [19] seasons have been previously published. Pasture mix paddocks were grazed hard with a commercial flock of mature age ewes after the early and late spring measurement periods to maintain the pasture quality by controlling reproductive stems and removing older leaves. Plantain mix and chicory mix paddocks were mowed at approximately 8 cm above soil level after the late spring season to remove ungrazed seed heads.

The lambs were commercially bought each seasonal study. The breed of lambs was Romney Cross and they were all cryptorchid male lambs. The age (months) and live weights (mean ± SEM kg) of the lambs at the start of each season were as follows: early spring (10, 38.1 ± 0.28), late spring/early summer (4, 32.6 ± 0.20), summer (4, 34.3 ± 0.20), and autumn (6, 34.2 ± 0.21) [19,20,21,22]. Each herbage treatment, within each season, had three groups of lambs serving as replicates. Each group was rotated around the same three paddocks within each treatment, during the experiment. Lambs were offered ad libitum intake with a herbage allowance of three times their predicted intake of 1.5 kg dry matter (DM) per head per day [23,24] by shifting the lambs into a new paddock when the post-grazing sward surface height reached 50 mm in the pasture mix and 70 mm in the plantain and chicory mixes [19,20,21,22]. In this experiment individual species height was not measured before commencing and after ending the season for each treatment. Instead the overall sward surface height was measured at pre- and post-grazing within each season.

The grazing area per herbage treatment was 2.25 ha consisting of three replicates of 0.75 ha arranged according to a randomized block design. Each replicate was subdivided into three 0.25 ha paddocks for rotational grazing. Lambs did not have any previous experience with plantain and chicory, but they had grazed ryegrass and white clover and potentially traces of red clover before being introduced to the experiment.

The herbage samples were taken from each paddock before and after grazing to determine the botanical composition for each season. Twelve herbage sample strips 200 mm long and 90 mm wide were cut from each paddock. After mixing all twelve samples together, a representative sample of 100 g was used to determine the botanical composition of each paddock (three per paddock) on a dry matter basis. The mean of the nine paddocks representing each treatment, per season, was considered as the botanical composition of the herbage treatment (pasture mix, plantain mix or chicory mix).

### 2.2. Transects

Twenty transects were placed within a paddock in each season for each herbage treatment giving a total of 200 tagged plants. Only one randomly selected paddock was used for each herbage treatment within each season (early spring, late spring, summer and autumn). Transects were set up by randomly placing pairs of wooden pegs two meters apart. Then a metal rod, which had been marked at 20 cm intervals, was placed between the two wooden pegs. Each mark was lettered from A to J. The plant that was nearest to each mark was tagged at its base using a coloured wire so it was clearly visible. There were ten tagged plants per transect reflecting the botanical composition in each transect.

Only the sown species within each herbage treatment were tagged. Hence, the pasture mix had only two tagged species; ryegrass and white clover. The plantain mix had three tagged species; plantain, white- and red-clover while, the chicory mix had four tagged species; plantain, chicory, white- and red-clover. Table 1 shows the percentage of tagged plants from each species within each season in each herbage treatment.

### 2.3. Animal Management

The stocking rates used for each season are given in Table 2. In each of the four seasons, paddocks containing the transects were grazed at both the beginning of the season and at the start of the second grazing rotation, when lambs returned to the same paddock again (on average, 22–29 days after the first grazing). Thus, there were two sets of transect readings per treatment for each season. At the first grazing within each season, but not the second, the herbs (plantain and chicory) were novel to the lambs.

Each recording period was for three consecutive days. Each tagged plant was checked at 24 h intervals to determine whether it had been eaten or not. If a leaf and/or leaves and/or reproductive material (flowers/panicles) and/or stem/s of the tagged plant were grazed partially or fully it was considered as ‘eaten’ on the first day this was observed. It was not checked again in that three day period. The outcome variable in this analysis was binary (0 or 1). If a tagged plant had been eaten it was given a notation of ‘1’ and if not eaten ‘0’. Day one data within each of the observed periods were used to interpret the proportional selection of different species on the first day of each period when the lambs were introduced to the respective treatment for the first time. The combined three days data were used to determine whether the lambs had eaten more of a given species over the entire three day period. The aim was to determine within each herbage treatment, the proportion of the total available plants within a given species that were selected for eating.

### 2.4. Statistical Analysis

#### 2.4.1. Botanical Composition

Each herbage treatment was analysed separately using proc GLM in SAS version 9.2 [25]. Botanical composition was analysed using a linear model with season and botanical composition at the start and at the end of the grazing period and the interaction between season as fixed effects. The means were separated using the LSD procedure in proc GLM.

#### 2.4.2. Selection Data within Herbage Treatment

Lamb selection of a given species (ryegrass or white clover or plantain or chicory or red clover) for a given season was defined as, the relative consumption of a species proportional to the number of tagged plants of that species.

The binary selection data (eaten or not), were statistically analysed, after logit transformation, separately for each herbage treatment (pasture mix, plantain mix or chicory mix) using Proc. GENMOD [25]. Different seasons (early spring or late spring or summer or autumn), species and the interaction between season × species were considered as fixed effects in the model. The same model was used for day 1 and combined three days data.

#### 2.4.3. Selection of White Clover across All Herbage Treatments

The selection of tagged white clover data (eaten or not) across different seasons and in the three herbage treatments were statistically analysed, after logit transformation using Proc. GENMOD [25]. Different seasons (early spring or late spring or summer or autumn), species and the interaction between season × species were considered as fixed effects in the model. The same model was used for day 1 and combined three days data.

## 3. Results

### 3.1. Botanical Composition

**Figure 2:**
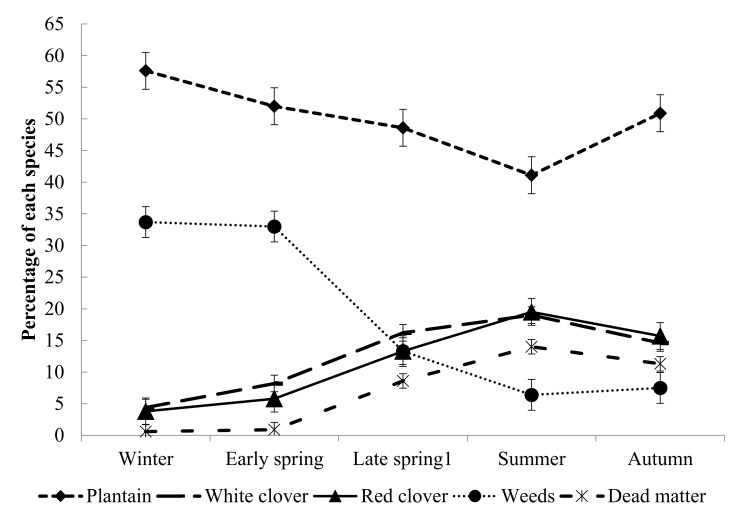
Botanical composition (%) within each season in the plantain mix. Late spring 1 and early summer. Vertical bars are ± SEM.

**Figure 3:**
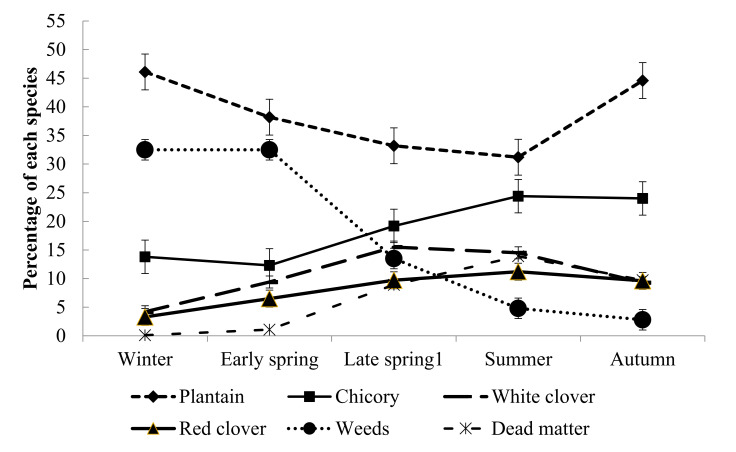
Botanical composition (%) within each season in the chicory mix. Late spring 1 and early summer. Vertical bars are ± SEM.

The proportion of different species in the plantain mix (Figure 2) and the chicory mix (Figure 3) differed across time. The proportion of plantain declined in late spring and summer in both the plantain and chicory mixes. The proportion of red clover in both herb-clover mixes and the proportion of chicory in the chicory mix increased during the late spring to summer season. The weed percentage in both herb-clover mixes decreased towards autumn.

### 3.2. Selection of Tagged Ryegrass and White Clover within the Pasture Mix

#### 3.2.1. Selection on Day One between Species

During early spring, lambs exhibited a greater (*p* < 0.05) selection of ryegrass compared to white clover (Table 3). However, during late spring, summer and autumn there was no difference (*p* > 0.05) in selection between the species.

#### 3.2.2. Within Species

Lambs exhibited a greater (*p* < 0.05) selection of ryegrass during early spring and autumn compared to late spring which in turn was greater (*p* < 0.05) than in summer. The selection of ryegrass during early spring and autumn did not differ (*p* > 0.05).

Lambs exhibited a greater (*p* < 0.05) selection of white clover during early spring, late spring and autumn than in summer. The selection of white clover during early spring, late spring and autumn did not differ (*p* > 0.05).

#### 3.2.3. Selection across the Combined Three Days between Species

In early spring, lambs exhibited a greater (*p* < 0.05) selection of ryegrass compared to white clover (Table 3). However, there was no difference between the two species (*p* > 0.05) in selection during any of the other seasons.

#### 3.2.4. Within Species

Lambs exhibited a greater (*p* < 0.05) selection of ryegrass during early spring than in late spring which in turn was greater (*p* < 0.05) than in autumn (Table 3). The selection of ryegrass was least (*p* < 0.05) during the summer season. There was a greater (*p* < 0.05) selection of white clover during early spring, late spring and autumn, than in summer (*p* < 0.05).

### 3.3. Selection of Tagged Plantain, White Clover and Red Clover within the Plantain Mix

#### 3.3.1. Selection on Day One between Species

Lambs exhibited no difference in selection for plantain, white clover or red clover during the early spring season (*p* > 0.05) (Table 4). During late spring, however, lambs exhibited a greater (*p* < 0.05) selection of red clover compared to either plantain or white clover, while the selection of both plantain and white clover did not differ (*p* > 0.05). During summer, lambs exhibited a greater (*p* < 0.05) selection of plantain and red clover compared to white clover. The selection of plantain and red clover did not differ (*p* > 0.05) in summer. There was no difference (*p* > 0.05) in the selection of any of the species during autumn.

#### 3.3.2. Within Species

Lambs exhibited a greater (*p* < 0.05) selection of plantain during summer compared to early spring which in turn was greater (*p* < 0.05) than in late spring (Table 4). The selection of plantain was lowest (*p* < 0.05) during autumn. There was no difference (*p* > 0.05) in selection of white clover across all the seasons. Lambs exhibited a greater (*p* < 0.05) selection of red clover during late spring and summer compared to early spring and autumn. There was no difference (*p* > 0.05) in selection of red clover between early spring and autumn. Similarly, during late spring and summer the selection of red clover did not differ (*p* > 0.05).

#### 3.3.3. Selection across the Combined Three Days between Species

In early spring, lambs exhibited a greater (*p* < 0.05) selection of plantain than white clover (Table 4). The selection of red clover did not differ (*p* > 0.05) from either plantain or white clover in early spring. There were no differences (*p* > 0.05) in the selection between species during late spring. During summer, lambs exhibited a greater (*p* < 0.05) selection of plantain and red clover than of white clover. The selection of plantain and red clover did not differ (*p* > 0.05) in summer. Lambs exhibited a greater (*p* < 0.05) selection of red clover than plantain and white clover during autumn. There was no difference (*p* > 0.05) in selection of either plantain or white clover during autumn.

### 3.4. Selection of Tagged Plantain, Chicory, White Clover and Red Clover within the Chicory Mix

#### 3.4.1. Selection on Day One between Species

Lambs did not exhibit (*p* > 0.05) any difference in selection for plantain, chicory, white clover or red clover during the early spring season (Table 5). During late spring, lambs exhibited a greater (*p* < 0.05) selection of red clover than plantain, chicory or white clover. The selection of plantain, chicory and white clover did not differ (*p* > 0.05) in late spring. During summer, lambs exhibited a greater (*p* < 0.05) selection of red clover than both plantain and chicory, which in turn were more selected (*p* < 0.05) than white clover. The selection of either plantain or chicory did not differ (*p* > 0.05) in the summer. During autumn, lambs exhibited a greater (*p* < 0.05) selection of red clover compared to both plantain and chicory. The selection of white clover was greater (*p* < 0.05) than chicory in autumn. The selection of plantain did not differ (*p* > 0.05) with either chicory or white clover in autumn. Similarly, the selection of red clover did not differ (*p* > 0.05) with white clover during autumn.

#### 3.4.2. Within Species

Lambs exhibited a greater (*p* < 0.05) selection of plantain during early spring, late spring and in summer than in autumn (Table 5). The selection during early spring, late spring and summer did not differ (*p* > 0.05). For chicory, lambs exhibited a greater (*p* < 0.05) selection during early spring, late spring and summer than in autumn. The selection of chicory was greater (*p* < 0.05) during summer than in late spring. The selection of chicory during early spring and summer did not differ (*p* > 0.05). For white clover, lambs exhibited a greater (*p* < 0.05) selection during early spring, late spring and in autumn than in summer. The selection during early spring, late spring and autumn did not differ (*p* > 0.05). Lambs exhibited a greater (*p* < 0.05) selection of red clover during late spring and in summer compared to autumn. The selection of red clover during late spring and summer did not differ (*p* > 0.05). The selection of red clover during early spring did not differ (*p* > 0.05) from selection in the other three seasons.

#### 3.4.3. Selection across the Combined Three Days between Species

Lambs did not exhibit (*p* > 0.05) any difference in selection of plantain, chicory, white clover or red clover during the early spring season (Table 5). Lambs exhibited a greater (*p* < 0.05) selection of plantain and white clover than chicory during late spring, but the selection of plantain and white clover did not differ (*p* > 0.05) during late spring. The selection of red clover did not differ (*p* > 0.05) from all the other herbage species in late spring. A greater (*p* < 0.05) selection of red clover was observed during summer than for both plantain and chicory, which in turn were greater (*p* < 0.05) than for white clover. The selection of plantain and chicory did not differ (*p* > 0.05) in summer. A greater (*p* < 0.05) selection of plantain and red clover was observed during autumn than chicory. The selection of plantain and red clover did not differ (*p* > 0.05) during autumn. The selection of white clover did not differ (*p* > 0.05) from the other herbage species during autumn.

#### 3.4.4. Within Species

Lambs exhibited a greater (*p* < 0.05) selection of plantain during late spring than in autumn (Table 5). The selection of plantain during early spring and summer did not differ (*p* > 0.05). Lambs exhibited a greater (*p* < 0.05) selection of chicory during summer than in late spring, which in turn was greater (*p* < 0.05) than in autumn. The selection of chicory during early spring was greater (*p* < 0.05) than in autumn but did not differ (*p* > 0.05) with other seasons. There was no difference (*p* > 0.05) in the selection of white clover across the different seasons. Lambs exhibited a greater (*p* < 0.05) selection of red clover during summer than in early spring, late spring and autumn. The selection of red clover during early spring, late spring or autumn did not differ (*p* > 0.05).

### 3.5. Selection of Tagged White Clover Plants across All the Seasons and Herbage Treatments

#### 3.5.1. Selection on Day One between Herbage Treatments

During early spring and summer both the plantain mix and chicory mix lambs displayed greater (*p* < 0.05) selection of white clover compared to the pasture mix lambs (Table 6). There was no difference (*p* > 0.05) in the selection of white clover by both plantain mix and chicory mix lambs during either early spring or summer. The plantain mix lambs displayed greater (*p* < 0.05) selection of white clover during late spring compared to the pasture mix lambs. The selection of white clover by the chicory mix lambs did not differ (*p* > 0.05) with the other two herbage treatments during late spring. There was no difference (*p* > 0.05) in the selection of white clover during autumn across treatments.

#### 3.5.2. Selection across the Combined Three Days between Herbage Treatments

During early spring, the selection of white clover by pasture mix, plantain mix and chicory mix lambs did not differ (*p* > 0.05 Table 6). During late spring and summer both plantain mix and chicory mix lambs exhibited a greater (*p* < 0.05) selection of white clover than pasture mix lambs. However, there was no difference (*p* > 0.05) in the selection of white clover by both plantain mix and chicory mix lambs during either late spring or summer. There was no difference (*p* > 0.05) in the selection of white clover during the early spring and autumn seasons between the herbage treatments.

## 4. Discussion

The relative abundance of the species in the herb-clover mixes was found to be maintained over two years when lambs were rotationally grazed using best practice rules [1,4], despite lambs exhibiting lower selection of plantain and chicory on the first day of grazing in autumn. As hypothesized, red clover was the species most selected within the herb-clover mixes by lambs, as previously observed by Pain et al. [11] and Cave et al. [12], but, unexpectedly, white clover was not favoured over the other species. The reason for the lower selection by lambs of white clover may be due to its lower accessibility in the pasture mix and the erect growth habit and abundant availability of plantain, chicory and red clover in the Plantain and chicory mixes [12].

In contrast to what was hypothesized, plantain in both the herb-clover mixes was selected similarly to the other species in all seasons, except autumn. In autumn on the first day of grazing, lambs selected fewer plantain, and chicory, plants than white- and red-clover plants. The cause of this shift in selection for plantain and chicory in autumn is unknown, but possible causes are low crude protein concentration and high concentrations of bioactives [9,14]. Low crude protein in plantain resulting from low soil moisture in summer has been shown to decrease sheep selection of plantain in early autumn [9,14]. The concentrations of the bioactives in plantain have been found to increase in summer and early autumn, and, although, implicated as a cause of decreased selection of plantain have not yet been shown to directly affect sheep selection. Although both plantain and chicory in the herb-clover mixes were eaten in the same proportion as the clover species at the completion of the three days of grazing in autumn, the low initial selection of the herbs could potentially affect the botanical composition by decreasing the proportion of clover species in the mix in the future if the pasture is frequently laxly grazed.

It was observed that the selection of red clover at day three was higher in summer and autumn than in early spring and late spring. While, red clover was the most selected species over time in both herb-clover mixes, as also observed by Pain et al. [11] and Cave et al. [12] for a chicory mix, the botanical composition data [22,26] suggested that the percentage of red clover in the mixes increased through summer and autumn. This increase in the percentage of red clover indicated that the proportional selection of red clover over the other species, plantain, chicory and white clover, did not negatively affect the red clover composition in the herb-clover mixes, in the short term at least.

The higher selection observed for both plantain and chicory in summer over white clover in the present experiment was in contrast to that observed by Cave et al. [12] in summer. There are multiple known factors that could contribute to increased selection of white clover and the consequent decrease in the percentage of white clover in the chicory mix and the plantain mix [26,27]. These factors include the greater accessibility of chicory and plantain due to their more erect nature than white clover. Previously, Cave et al. [12] suggested that the lower availability of white clover in the upper strata of a herb-clover mix may have resulted in a poor selection of white clover during the late spring season. In addition, the chicory mix paddocks were mowed during summer to remove chicory seed heads and to maintain vegetative growth, which is standard management [4,28], offering lambs good quality leafy herbage of both the chicory and plantain in this mix. This mowing might also increase selection of chicory and plantain in summer over white clover. Other factors are the superior drought tolerance of plantain, chicory and red clover compared to white clover [29], and the selective grazing by lambs decreasing the persistence of white clover [30]. Overall, it appears that it is likely to be more difficult to maintain the abundance of white clover than the other species in herb-clover mixes.

White clover was the only species in all three of the herbage treatments, and so provided the opportunity to test if selection by lambs of white clover was different in any of the herbage treatments. In the plantain mix and chicory mix, lambs proportionally grazed more white clover on day one compared to the pasture mix lambs in the early spring to summer season. This may have been due to white clover being more abundant and available in these herb-clover mixes compared to the pasture mix.

After the combined three days of grazing, in early spring, late spring, summer and autumn, between 73 to 100% of white clover plants in the plantain and chicory mixes were grazed. Whereas in the pasture mix during the summer season only 44% of the white clover was grazed, possibly due to the high percentage of ryegrass dead matter in the sward, in response to the higher temperature and lower soil moisture in summer, restricting access. The low selection by lambs of both white clover and ryegrass in the pasture mix during summer compared with their high selection of all the species in the herb-clover mixes suggested the pasture mix was less palatable in summer than the herb-clovers mixes. The superior feeding value of the two herb-clover mixes in summer was also supported by their stocking rate being 1.7 times greater, and their lamb live weight gain being 1.5 times greater as reported by Somasiri et al. [22], than for the pasture mix. [22]. Additionally, Kenyon et al. [31] showed that over three summer seasons the average daily live weight gain of lambs grazing either a plantain mix or a chicory mix was 1.7 times that for lambs grazing a pasture mix.

## 5. Conclusions

Lamb diet selection does not affect the relative abundance of species in herb-clover mixes over at least two years despite lower selection of plantain and chicory on the first day of grazing in autumn. Consequently, the superior lamb live weight gains of these herb-clover mixes, relative to ryegrass and white clover, can be readily maintained with standard grazing management.

## Figures and Tables

**Table 1:** Percentages of tagged plant species in the herbage treatments (pasture mix, plantain mix and chicory mix) at the start of each grazing season. Ryegrass (Rye), white clover (WC), plantain (Plan), red clover (RC), chicory (Chic).

Season	Percentages (%)								
	Pasture Mix		Plantain Mix			Chicory Mix			
	Rye	WC	Plan	WC	RC	Plan	Chic	WC	RC
Early spring	86.5	13.5	55.0	17.5	27.5	58.5	27.5	7.0	7.0
Late spring	88.0	12.0	69.0	20.5	10.5	51.0	24.0	11.0	14.0
Summer	73.5	26.5	60.0	20.0	20.0	39.5	33.0	12.0	15.5
Autumn	91.0	9.0	76.0	8.5	15.5	42.5	28.0	8.0	21.5

**Table 2:** Stocking rates (no. of sheep/ha) used in each season.

Treatment	Early Spring	Late Spring	Summer	Autumn
Pasture mix	30	40	24	28
Plantain mix	30	40	40	32
Chicory mix	25	40	40	32

**Table 3:** Proportional selection by the pasture mix lambs of tagged ryegrass and tagged white clover on day one and three of grazing a plot during different seasons (logit lsmeans ± SEM, back transformed percentages (%) are given in brackets).

Day	Season	Species		Level of Significance		
		Ryegrass	White Clover	Species	Season	Species × Season
One	Early spring ^1^	−0.28 ^b z^ ± 0.11 (43)	−1.25 ^a y^ ± 0.33 (22)	<0.001	<0.001	<0.05
	Late spring	−1.24 ^y^ ± 0.13 (23)	−0.72 ^y^ ± 0.30 (33)			
	Summer	−3.46 ^x^ ± 0.34 (3)	−4.65 ^x^ ± 1.00 (1)			
	Autumn	−0.28 ^z^ ± 0.11 (43)	−0.34 ^y^ ± 0.34 (42)			
Three	Early spring ^2^	2.09 ^b z^ ± 0.17 (89)	1.15 ^a y^ ± 0.32 (76)	<0.01	<0.001	>0.05
	Late spring	1.23 ^y^ ± 0.14 (81)	1.49 ^y^ ± 0.37 (82)			
	Summer	−0.26 ^w^ ± 0.12 (44)	−0.23 ^x^ ± 0.20 (44)			
	Autumn	1.07 ^x^ ± 0.12 (74)	1.61 ^y^ ± 0.45 (83)			

^1^ Within the season interaction given by differing superscripts (a, b) within rows indicates significant difference (*p* < 0.05). ^2^ Between season interaction given by differing superscripts (w, x, y, z) within columns indicates significant difference (*p* < 0.05).

**Table 4:** Proportional selection by the plantain mix lambs of a tagged species, plantain, white clover and red clover, on day one and three during different seasons (logit lsmeans ± SEM, back transformed percentages (%) are given in brackets).

Day	Season	Species			Level of Significance		
		Plantain	White Clover	Red Clover	Species	Season	Species × Season
One	Early spring ^1^	0.21 ^y^ ± 0.13 (55)	−0.03 ± 0.24 (49)	0.03 ^x^ ± 0.23 (51)	<0.01	<0.001	<0.001
	Late spring	−0.17 ^a x^ ± 0.12 (46)	0.10 ^a^ ± 0.22 (52)	1.16 ^b y^ ± 0.36 (76)			
	Summer	0.58 ^b z^ ± 0.13 (64)	−0.46 ^a^ ± 0.23 (39)	1.12^b y^ ± 0.26 (75)			
	Autumn	−0.91 ^w^ ± 0.13 (29)	−0.43 ± 0.36 (39)	−0.58 ^x^ ± 0.26 (36)			
Three	Early spring ^2^	2.56 ^b y^ ± 0.24 (93)	1.46 ^a x^ ± 0.31 (81)	1.93 ^ab x^ ± 0.34 (87)	>0.05	<0.001	<0.001
	Late spring	2.66 ^y^ ± 0.24 (93)	0.96 ^y^ ± 0.59 (96)	3.71 ^xy^ ± 1.01 (98)			
	Summer	3.85 ^b z^ ± 0.45 (98)	2.07 ^a xy^ ± 0.35 (89)	4.38 ^b y^ ± 1.01 (99)			
	Autumn	1.24 ^a x^ ± 0.14 (78)	0.98 ^a x^ ± 0.39 (73)	2.10 ^b x^ ± 0.40 (89)			

^1^ Within the season interaction given by differing superscripts (a, b) within rows indicates significant difference (*p* < 0.05). ^2^ Between season interaction given by differing superscripts (w, x, y, z) within columns indicates significant difference (*p* < 0.05).

**Table 5:** Proportional selection by the chicory mix lambs of a tagged species, plantain, chicory, white clover and red clover on day one and three during different seasons (logit lsmeans ± SEM, back transformed percentages (%) are given in brackets).

Day	Season	Species				Level of Significance		
		Plantain	Chicory	White Clover	Red Clover	Species	Season	Species × Season
One	Early spring ^1^	0.27 ^y^ ± 0.13 (57)	0.41 ^yz^ ± 0.19 (60)	0.80 ^y^ ± 0.40 (69)	0.75 ^xy^ ± 0.40 (68)	<0.001	<0.001	<0.001
	Late spring	0.27 ^a y^ ± 0.14 (57)	−0.02 ^a y^ ± 0.20 (49)	−0.00 ^a y^ ± 0.30 (50)	1.30 ^b y^ ± 0.33 (79)			
	Summer	0.60 ^b y^ ± 0.17 (65)	0.91 ^b z^ ± 0.19 (71)	−1.10 ^a x^ ± 0.33 (25)	1.77 ^c y^ ± 0.36 (85)			
	Autumn	−0.25 ^ab x^ ± 0.16 (44)	−0.56 ^a x^ ± 0.20 (36)	0.25 ^bc y^ ± 0.36 (56)	0.42 ^c x^ ± 0.22 (60)			
Three	Early spring ^2^	3.21 ^xy^ ± 0.34 (96)	3.57 ^y z^ ± 0.59 (97)	2.60 ± 0.73 (93)	2.56 ^x^ ± 0.73 (93)	<0.001	<0.001	<0.001
	Late spring	4.20 ^b y^ ± 0.58 (99)	2.41 ^a y^ ± 0.37 (92)	26.37 ^b^ ± 0.93 (100)	4.01 ^ab x^ ± 1.01 (98)			
	Summer	3.42 ^b xy^ ± 0.45 (97)	3.76 ^b z^ ± 0.58 (98)	1.47 ^a^ ± 0.37 (81)	26.37 ^c y^ ± 1.01 (100)			
	Autumn	2.48 ^b x^ ± 0.29 (92)	1.48 ^a x^ ± 0.24 (81)	2.27 ^ab^ ± 0.61 (91)	3.74 ^b x^ ± 0.72 (98)			

^1^ Within the season interaction given by differing superscripts (a, b) within rows indicates significant difference (*p* < 0.05). ^2^ Between season interaction given by differing superscripts (w, x, y, z) within columns indicates significant difference (*p* < 0.05).

**Table 6:** Proportional selection of tagged white clover by lambs across all the seasons between treatments (pasture mix vs. plantain mix vs. chicory mix) (logit lsmeans ± SEM, back transformed percentages (%) are given in bracket).

Day	Season	Treatment			Level of Significance		
		Pasture Mix	Plantain Mix	Chicory Mix	Season	Treatment	Season × Treatment
**One**	Early spring ^1^	−1.25 ^a y^ ± 0.33 (22)	−0.03 ^b^ ± 0.24 (49)	0.80 ^b y^ ± 0.40 (69)	<0.001	<0.001	<0.001
	Late spring	−0.72 ^a y^ ± 0.30 (33)	0.10 ^b^ ± 0.22 (52)	0.00 ^ab y^ ± 0.30 (50)			
	Summer	−4.65 ^a x^ ± 1.00 (1)	−0.46 ^b^ ± 0.23 (39)	−1.10 ^b x^ ± 0.33 (25)			
	Autumn	−0.34 ^y^ ± 0.34 (42)	−0.43 ± 0.36 (39)	0.25 ^y^ ± 0.36 (56)			
Three	Early spring ^2^	1.15 ^y^ ± 0.32 (76)	1.45 ^x^ ± 0.31 (81)	2.60 ^x^ ± 0.73 (93)	<0.001	<0.001	<0.001
	Late spring	1.49 ^a y^ ± 0.37 (82)	3.27 ^b y^ ± 0.59 (96)	25.37 ^b y^ ± 0.93 (100)			
	Summer	−0.23 ^a x^ ± 0.20 (44)	2.07 ^b xy^ ± 0.35 (89)	1.47 ^b x^ ± 0.37 (81)			
	Autumn	1.61 ^y^ ± 0.45 (83)	0.98 ^x^ ± 0.39 (73)	2.27 ^x^ ± 0.61 (91)			

^1^ Within the season interaction given by differing superscripts (a, b) within rows indicates significant difference (*p* < 0.05). ^2^ Between season interaction given by differing superscripts (x, y) within columns indicates significant difference (*p* < 0.05).
